# Alternative Splicing of a Novel Inducible Exon Diversifies the CASK Guanylate Kinase Domain

**DOI:** 10.1155/2012/816237

**Published:** 2012-09-12

**Authors:** Jill A. Dembowski, Ping An, Maritsa Scoulos-Hanson, Gene Yeo, Joonhee Han, Xiang-Dong Fu, Paula J. Grabowski

**Affiliations:** ^1^Department of Biological Sciences, University of Pittsburgh, Pittsburgh, PA 15260, USA; ^2^Department of Cellular and Molecular Medicine, Institute for Genomic Medicine, University of California, San Diego, La Jolla, CA 92093, USA; ^3^Stem Cell Program, University of California, San Diego, La Jolla, CA 92093, USA

## Abstract

Alternative pre-mRNA splicing has a major impact on cellular functions and development with the potential to fine-tune cellular localization, posttranslational modification, interaction properties, and expression levels of cognate proteins. The plasticity of regulation sets the stage for cells to adjust the relative levels of spliced mRNA isoforms in response to stress or stimulation. As part of an exon profiling analysis of mouse cortical neurons stimulated with high KCl to induce membrane depolarization, we detected a previously unrecognized exon (E24a) of the CASK gene, which encodes for a conserved peptide insertion in the guanylate kinase interaction domain. Comparative sequence analysis shows that E24a appeared selectively in mammalian CASK genes as part of a >3,000 base pair intron insertion. We demonstrate that a combination of a naturally defective 5′ splice site and negative regulation by several splicing factors, including SC35 (SRSF2) and ASF/SF2 (SRSF1), drives E24a skipping in most cell types. However, this negative regulation is countered with an observed increase in E24a inclusion after neuronal stimulation and NMDA receptor signaling. Taken together, E24a is typically a skipped exon, which awakens during neuronal stimulation with the potential to diversify the protein interaction properties of the CASK polypeptide.

## 1. Introduction

Alternative pre-mRNA splicing generates protein diversity throughout the transcriptome, whereas mistakes in its regulation underlie a variety of human diseases [1, 2]. Mechanisms of exon skipping and inclusion, as well as 5′ and 3′ splice site selection, are commonly used to produce multiple mRNA isoforms from a single gene. Regulation occurs during the dynamic stages of spliceosome assembly and involves the interactions of small nuclear ribonucleoprotein complexes (snRNPs) and numerous protein factors, such as SR, hnRNP, and KH-type splicing factors, with the pre-mRNA [3, 4].

Internal cassette exons are recognized through interactions at the 5′ splice site with U1 followed by U6 snRNP, together with interactions at the branch site/3′ splice site region with U2 snRNP and U2 auxiliary factor (U2AF). Members of the SR protein family contribute essential roles as enhancers of exon recognition when the splice sites are less than ideal, which is typically the case for mammals. Their modular protein structures allow for the simultaneous recognition of exonic RNA sequence motifs (via the N-terminal RNA-binding domain) and components of U1 snRNP and/or U2AF bound to their respective splice sites (via the C-terminal RS-dipeptide-enriched domain) [5]. SR proteins can also function as splicing silencers to promote exon skipping depending on the location of their binding sites in the pre-mRNA [6].

Proteins of the hnRNP families play important roles in the regulation of exon skipping (silencing) or inclusion (enhancing) patterns. Their silencing and enhancing roles depend upon the RNA code or map of the relative positions of their binding sites in the pre-mRNA and whether or not their functions antagonize or promote those of the core snRNPs. Tissue and developmental stage-specific expression and modification of these factors modulate splicing changes in a spatial and temporal manner. The brain-enriched splicing factor, Nova, recognizes YCAY (Y, pyrimidine) core elements to regulate the splicing of transcripts that encode protein components of the synapse [7, 8]. The forebrain-enriched factor, CUGBP2, regulates the production of NMDA receptor isoforms and through the recognition of UGUGU core and UGU auxiliary motifs at the perimeters of the branch sites directs exon skipping [9]. In addition, the polypyrimidine tract-binding protein (PTB) and the related nPTB regulate splicing events that are important for normal brain development through the recognition of UCUU and related core motifs [10].

The field is at an early stage of understanding the splicing codes responsible for the specification of cell-type, developmental stage-specific, and responsive alternative splicing due to the intricate nature of the mechanisms involved [11–13]. Sequence elements have been identified that mediate inducible exon-skipping events in the CD45 transcript by hnRNP LL in activated T cells [14, 15]. Calcium calmodulin kinase (CaMK) responsive RNA elements I and II (CaRRE I and II) have also been shown to mediate inducible exon skipping of the STREX exon through pathways involving CaMK IV [16, 17]. Roles for UAGG motifs, splicing factor hnRNP A1, and functional NMDA receptors have also been implicated in the inducible skipping of E19 of the NMDA R1 transcript in cortical neurons stimulated with high KCl [18]. While these studies highlight insights into mechanisms of inducible exon skipping, comparatively less is known about inducible exon inclusion.

The phenomenon of splicing responsiveness suggests that cells take advantage of the flexibility of splicing to adjust their protein activities for survival or adaptation. For example, alternative splicing can be used to tune protein localization or protein-protein interaction properties of a polypeptide [19]. Furthermore, groups of proteins that are regulated by a particular splicing factor may physically interact or may be linked through functional networks [8, 20].

In this study we identify a novel skipped exon of the calcium/calmodulin-dependent serine protein kinase (CASK) transcript, which is selectively included in response to neuronal excitation. This exon—termed E24a—is not annotated as an exon in the Ensembl database for any of the 17 spliced variants of the mouse CASK gene. CASK is a synaptic scaffolding protein with multiple interaction domains characteristic of the membrane-associated guanylate kinase (MAGUK) family. These include an N-terminal calcium/calmodulin kinase domain, two L27 domains, PDZ and SH3 domains, and a C-terminal guanylate kinase domain. CASK is enriched at the neuronal synapse where it plays important roles in regulating trafficking, targeting, and signaling of ion channels [21, 22]. During development, CASK is also recruited into the nucleus where it is involved in the regulation of neuronal gene expression.

Here we use comparative sequence analysis and splicing reporter assays to explore the phylogenetic distribution and splicing factors affecting regulation of the E24a coding exon. Based on the unusual splicing responsiveness of the exon in the context of a cohort of 24 unresponsive and differentially regulated exons of the endogenous CASK gene, we identify the noncanonical 5′ splice site of E24a and clusters of neighboring SC35 motifs as distinguishing characteristics. We show evidence of a strong silencing role for SC35 and the noncanonical 5′ splice site of E24a, which together reinforce the exon-skipping mechanism. Furthermore, we demonstrate that E24a is subject to negative regulation by SF2/ASF, Nova, PTB, CUGBP2, and hnRNP H, as well as positive regulation by the neural enriched SR protein, SRp75. These results are consistent with the local arrangement of silencing motifs in a highly conserved sequence block containing E24a. By all appearances, these results suggest a model in which E24a is in the midst of an evolutionary transition from that of a pseudoexon harbored in an intron sequence and that of a functional coding exon emerging with the potential to diversify the properties of the CASK polypeptide when activated by cellular stimulation.

## 2. Materials and Methods

### 2.1. Cortical Neurons, KCl Stimulation, and RNA Analysis

One dozen mouse (CD1 strain) embryonic day 18 cerebral cortex tissues (Hilltop Lab Animals, Inc) were dissociated and plated on poly-D-lysine coated 10 cm dishes (BD Biosciences) as described [18]. Neurons were grown in Neurobasal media with B27 supplement and 0.5 mM Glutamax (Invitrogen) for 10 days in culture. KCl treatment was carried out for 16 hours at a final concentration of 50 mM. Mock treatment was performed in parallel without KCl addition. Treatment with NMDA receptor antagonists, MK801 and AP5 (Sigma Aldrich), was carried out by adding AP5 to 100 *μ*M and MK801 to 25 *μ*M to each 10 cm dish together with KCl. RNA was harvested by the addition of Trizol (3 mL for each 10 cm plate) and quantified by A_260_ measurement. mRNA was further purified on RNeasy columns (Qiagen). RNA samples used for RT-PCR validation were the same as those used for microarray analysis. Primer pairs specific for the flanking exons were designed based on exon coordinates provided by the microarrays using sequence information on Ensembl and UCSC genome browsers. Primer sequences are available upon request. Reaction products were detected by ethidium bromide staining after separation on 1.5–2% agarose gels. For sequence confirmation, both the exon included and skipped DNA products of the PCR reactions were gel purified and cloned into pBS-SK vector by blunt end ligation. Constructs containing single inserts were chosen for DNA sequencing (Genewiz).

### 2.2. Splicing Reporter Assays

The splicing reporter minigene for CASK E24a was generated by PCR amplifying a genomic DNA fragment including CASK E24a and surrounding intron sequences (235 bp upstream, 251 bp downstream). Purified PCR products were digested with *Nde*I and *Xba*I followed by gel purification and then ligated into pA+SIRT1a vector backbone [18] cut with the same restriction enzymes. The DIP5pss splicing reporter was generated from the DIP13 E5 splicing reporter [18] using the QuikChange Site-Directed Mutagenesis Kit (Stratagene). Positive clones were verified by restriction digestion and DNA sequencing (Genewiz). Protein expression plasmids for hnRNP H [23], PTB [24], CUGBP2 [25], Nova [26], hnRNP L [27], SRp75 [28], SC35, and ASF/SF2 [29] were as described previously. Mouse neuroblastoma (N18TG2) and myoblast (C2C12) cells were maintained in Dulbecco's modification of Eagle's medium (DMEM) supplemented with 10% fetal bovine serum (FBS). Lipofectamine 2000 (Invitrogen) was used for transfections in 6-well plates at a cell density of 60%. For each transfection, a total of 1.25 *μ*g of DNA was used, including 0.25 *μ*g of splicing reporter and 1.0 *μ*g of protein expression plasmid or vector backbone. Transfections were performed in 2.5 *μ*L of lipofectamine 2000 following the manufacturer's instructions. Total RNA was harvested from the cells with Trizol (Invitrogen) at 20 to 24 hours following transfection and analyzed by RT-PCR as described [23]. Expression from protein expression plasmids was verified by Western blot analysis of cell lysates from transfected cells.

### 2.3. Inducible SC35 Expression in MEF Cells

MEF cells were cultured in 6-well plates to a confluency of ~50–60% in the presence or absence of Doxycycline (Dox) for 2 days and transfected with 0.67 *μ*g of splicing reporter plasmid in serum-free medium at 37°C for 4 hours. After 4 hours of transfection, the medium was replaced by DMEM (-Dox) with 10% FBS and antibiotics, and the MEFs were cultured for 48 hours without or with 10 *μ*g/mL Dox. Under these conditions exogenous HA-tagged SR proteins were depleted by the addition of Dox. Total RNA was extracted from the MEFs using Trizol, and 1 *μ*g of the total RNA was used to synthesize cDNA with Superscript III (Invitrogen) according to the manufacturer's instructions at the concentrations indicated using random hexamer primers. PCR was performed using 1 *μ*L of the cDNA in 50 *μ*L reactions with TaqGold (Roche), and products were analyzed on 2% agarose gels.

### 2.4. Sequence Motif Analysis

Intron/exon sequences were obtained for analysis from the Ensembl database (http://www.ensembl.org/). The frequency of trinucleotides across the CASK gene was determined with MacVector base composition analysis (Accelrys). ESE motifs were identified using ESEFinder 3.0 program (http://rulai.cshl.edu/tools/ESE3/). MAXENT splice site scores were determined using the MaxEntScan program (http://genes.mit.edu/burgelab/maxent). Sequence alignments were performed using ClustalW (http://www.ebi.ac.uk/Tools/). Modeling of the polypeptide was carried out using MUSTER (http://zhanglab.ccmb.med.umich.edu/MUSTER/).

## 3. Results

**A Novel Responsive Exon Encodes a Peptide Insertion in the Guanylate Kinase Domain of the CASK Polypeptide**

**Figure 1:**
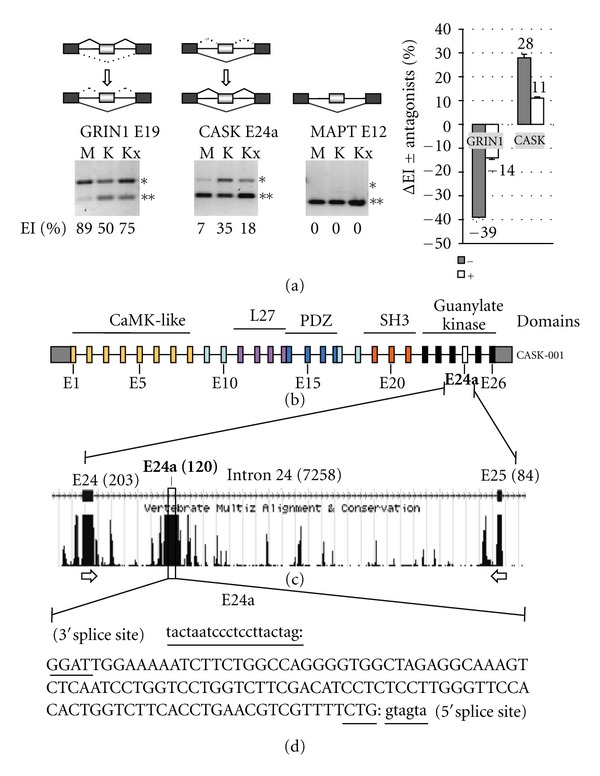
Identification of a highly conserved, responsive exon of the mouse CASK transcript. (a) RNA samples used for RT-PCR validation of splicing pattern changes were derived from cortical cultures following mock treatment (lanes M) or stimulation with high KCl (lanes K). Additional samples were supplemented with MK801 and APV to attenuate the splicing response (lanes Kx). Gel panels illustrate the exon included (*) and skipped (**) products amplified by primers specific for the flanking exons of GRIN1 E19 (left panel), CASK E24a (middle panel), and MAPT E12 (right panel). Numbers at bottom of gel panels represent the % exon inclusion (%EI) values for each lane. The schematics above summarize the observed changes in exon inclusion for E19 and E24a and the unresponsive exon-skipping pattern of E12. Graph at right shows the change in exon inclusion (ΔEI) values for E19 and E24a as a function of stimulation with (white bars, +) and without (grey bars, −) MK801 and AP5. (b) The exon-intron structure of the mouse CASK gene is shown using the exon-numbering scheme from the Ensembl CASK-001 transcript; corresponding protein domains (color-coded) are shown above. The position of E24a (white box) as identified in this study is shown. (c) Expanded region shows the sequence conservation of E24a (Multiz Alignment) and its position in the context of the full-length intron 24. Exon/intron sizes (base pairs) are indicated in parentheses. Note that the sequence of mouse E24a corresponds to coordinates 12,688,825–12,688,945 on the X chromosome from UCSC Genome Browser, 2006 genome assembly. Arrows indicate primers used for the RT-PCR analysis of (a). (d) The confirmed sequence of E24a (uppercase) and adjacent 5′ and 3′-splice sites (underscored) are shown. Colons indicate exon/intron boundaries.

The observation initiating this study was the detection of a candidate responsive exon within the mouse CASK transcript, which was enriched in neuronal cultures treated with high KCl to induce membrane depolarization (KCl stimulated), relative to control samples (mock). These results were part of a larger analysis using Affymetrix exon arrays, which will not be reported here. To test the validity of the splicing pattern change indicated for CASK by the microarray experiments, we performed RT-PCR analysis with the original set of RNA samples using primers specific for the flanking exons, E24 and E25, of the mouse CASK-001 transcript. These results confirmed a substantial increase for middle exon inclusion for the KCl stimulated, relative to the mock-treated samples (Figure 1(a), CASK E24a panel, lanes M, K). Control reactions illustrate the splicing pattern change for a known inducible exon, E19 of the mouse GRIN1-004 transcript, which undergoes exon skipping in these samples (GRIN1 panel, lanes M, K). E12 of Microtubule-Associated Protein Tau (MAPT) is representative of an unresponsive skipped exon in this analysis, which indicates that KCl stimulation does not uniformly enhance splicing efficiency for a skipped exon (MAPT panel, lanes M, K).

To assess the potential biological relevance of the CASK splicing pattern change a third culture sample was treated with high KCl in the presence of NMDA receptor-specific antagonists, which are known to reduce the exon-skipping response of GRIN1 E19 in neurons resulting from the blockage of calcium influx [18]. These results show that the change in exon inclusion for E24a under high KCl conditions is reduced in the presence of the antagonists as for E19 (Figure 1(a), lanes Kx and graph). Thus, the splicing pattern change observed for the CASK transcript is consistent with a biological response mediated by functional NMDA receptors.

These results indicated that the inducible exon was coming from a 7,258-nucleotide intron (intron 24) within the guanylate kinase-coding region of the CASK transcript (Figure 1(b)), but supporting EST and cDNA evidence for an exon in this position of the transcript was lacking. This uncertainty prompted us to clone and sequence the E24a included (E24-E24a-E25) and skipped (E24-E25) products from the RT-PCR experiments. The confirmed sequence of E24a matched exactly to a highly conserved, 120 nucleotide sequence towards the beginning of intron 24 of the Ensembl CASK-001 transcript (Figures 1(c) and 1(d)). Consistent with its designation as an exon, intact 3′ and 5′ splice sites were found at the boundaries of E24a (Figure 1(d), underlined). Of the 17 spliced variants of mouse CASK in the Ensembl database, none of these annotated exons matched the E24a sequence. Note that E24a is not related to a previously identified brain-included exon of CASK [30].

We next searched for the E24a nucleotide sequence in corresponding introns of orthologous CASK genes and assessed these for peptide coding potential. Genomic sequences taken from the Ensembl database were used for nucleotide sequence alignments to identify E24a sequences for conceptual translation. Intron 24, which harbors E24a in mouse CASK, was consistently found in the intron between highly conserved exons of lengths 203 and 84 base pairs, respectively, for horse, dolphin, cow, mouse, rat, chimp, human, and armadillo (Table 1). In these mammalian species, the position of E24a was consistently towards the beginning of the intron, and conceptual translation showed coding potential for these E24a's continuous with the flanking exons as was found for mouse CASK. In contrast, the E24a sequence was not found in the CASK genes of lower vertebrates, such as lizard, chicken, fugu, xenopus, and zebrafish, each of which had a much smaller intron (<2900 base pairs) between the highly conserved (203 and 84 base pair) flanking exons. Thus, E24a is a novel exon conserved in mammals, and its inclusion by splicing predicts the corresponding insertion of a 40 amino acid peptide in the guanylate kinase domain of the CASK polypeptide.

**Three Cassette Exons of CASK Show Divergent Responses to KCl Stimulation Despite Similar Trends in Their Splicing Patterns During Development**

**Figure 2:**
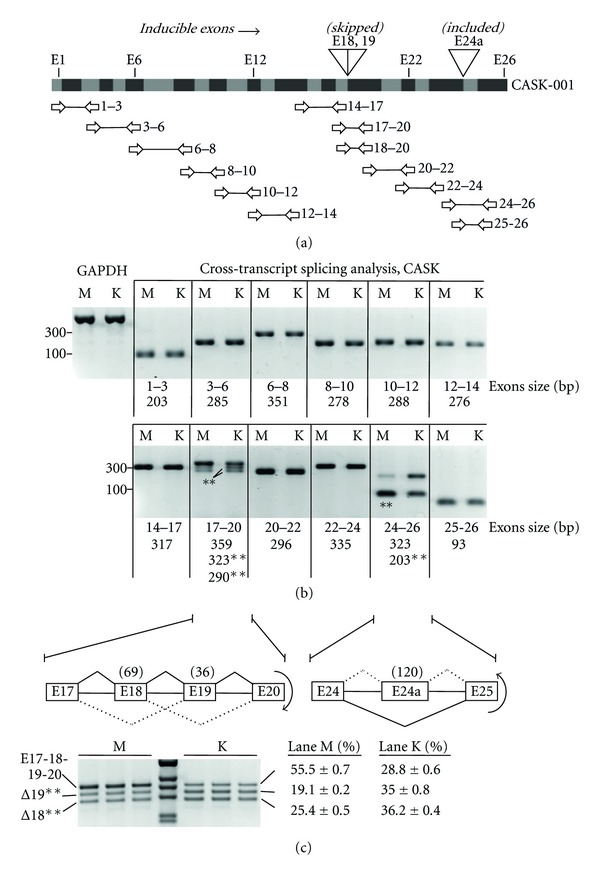
Splicing analysis across the CASK gene reveals three alternative exons with divergent responses to neuronal excitation. (a) Schematic (top) illustrates CASK-001 mRNA with consecutive exons represented as alternating grey and black boxes. Inducible exons identified in this study (E18, E19, E24a) are highlighted above. Horizontal arrows represent primer sets used for RT-PCR amplification of regions spanning the exon numbers at right. (b) Gel panels show results of RT-PCR to detect splicing changes from mock (lanes M) and KCl-treated (lanes K) mouse cortical neurons. Exon regions corresponding to (a) are indicated below the gel panels, and sizes of the amplified products are indicated in base pairs (bp). Asterisks (**) indicate skipped products; unmarked bands represent included products. GAPDH transcripts were amplified as controls (lanes GAPDH). Fragment sizes, 300 and 100 bp (at left), were determined from a 100 kb DNA ladder. (c) Higher resolution analysis of the E18 and 19 region. Schematics represent initial splicing pattern and inducible change (curved arrows) for alternative exons of CASK. Numbers in parentheses represent exon sizes in base pairs. Gel panel shows triplicate measurements of the E17-18-19-20, the Δ19 and Δ18 spliced products amplified from mock (M) and KCl stimulation (K) samples. Numbers represent average % exon inclusion and standard deviations from mock (left column) and KCl (right column) datasets.

To understand whether the inducible effects on the splicing of E24a represent indiscriminate effects or a more selective form of splicing regulation in the context of the endogenous gene, splicing patterns were analyzed throughout the length of the mouse CASK transcript using a series of nested PCR primer pairs (Figure 2(a)). This analysis was designed to test more broadly for exon inclusion or skipping patterns for 24 internal exons and for potential cryptic exons that, like E24a, might be harbored within intron segments. The expected product sizes were in the ~300 base pair range, which should reveal alternative exons as small as 20–50 base pairs in our system. This assay revealed additional splicing changes in the E17–E20 region but was otherwise negative for splicing changes outside of this and the E24a regions of the CASK transcript (Figure 2(b)). Higher resolution separation of the spliced products verified that the changes in the E17–20 region involved inducible exon skipping of two adjacent cassette exons, E18 and E19 (Figure 2(c)). These results showed that the spliced product containing both E18 and E19 decreased proportionately as the individual spliced forms lacking either E18 or E19 increased. A product lacking both exons was not observed. Note that the identities of E18 and E19 as the inducible skipped exons were confirmed by sequencing of the reaction products. Thus, only 3 of 25 internal exons of the CASK transcript behave as inducible exons under these conditions indicating that KCl stimulation mediates selective changes to these regulatory mechanisms.

Notably within the CASK transcript, E18 and E19 showed inducible exon skipping under the same conditions of KCl stimulation that induced E24a inclusion. Because of this divergent behavior, we wished to calibrate the KCl-induced splicing changes of these exons—measured in dissociated cortical neurons in culture—against any physiological changes occurring during development in the intact tissue. Three time points spanning embryonic day 18 to postnatal day 21 of the mouse cerebral cortex were chosen to represent a significant period of development for this brain region. Whereas the KCl-induced exon-skipping behavior of E18 and E19 showed good agreement with the trend observed during development, the E24a-included spliced variant did not. That is, E24a showed strikingly different behavior during KCl stimulation relative to development both with respect to the trend and the extent of the splicing change (see Supplementary Figure S1 available online at doi: 10.1155/2012/816237). The E24a-included variant was at its highest level at embryonic day 18 (15%), and this variant gradually decreased during development (8%). Here, KCl stimulation increased E24a inclusion in opposition to the decrease observed during development, and the extent of the change was higher for the KCl-stimulated samples (difference of +28% for KCl stimulated versus −7% for development). These data indicate that, unlike E18 and E19, KCl stimulation induces a splicing change for E24a not representative of that observed in this window of development in the cerebral cortex. Therefore, E24a inclusion is likely a consequence of a specific physiological change in the splicing machinery that selectively regulates this exon after membrane depolarization.

**Skipping of E24a is Associated with a Weak Noncanonical 5′ Splice Site**

**Figure 3:**
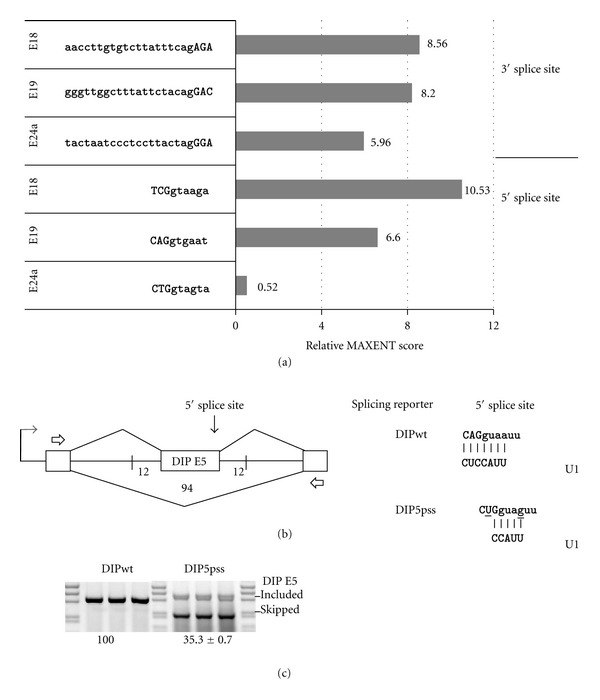
A noncanonical 5′ splice site represents an unusual feature of E24a, and transplantation of this splice site renders a constitutive exon susceptible to splicing silencing. (a) Graph illustrates relative splice site scores determined using the MAXENT scoring algorithm for E18, E19, and E24a. Sequences used for scoring are shown. (b) Splicing reporter, DIPwt, contains constitutive exon, E3, as the middle exon with 12 bases of flanking intron sequence. E3 and adjacent intron sequences are derived from the mouse DIP13beta (APPL2) gene. Sequences of the 5′ splice sites of DIPwt and DIP5pss are shown at right (5′ to 3′); vertical lines illustrate potential base pairing to U1 snRNA (3′ to 5′). The 5′ splice site of CASK E24a was transferred into DIP5pss by constructing two-point mutations (underscored). (c) The gel panel shows results of splicing assays performed after transfecting DIPwt and DIP5pss reporters into N18TG2 cells. Transfections and RT-PCR amplification were carried out in triplicate. Numbers below gel panels represent average % exon inclusion value ± standard deviation.

Analysis of tissue-specific inclusion of E24a demonstrated that this exon is skipped in most tissue types (skeletal muscle, heart, liver, thymus, lung, ovary, and testes) but is weakly included in the nervous system (brain, spinal cord, cerebral cortex, hippocampus, cerebellum, and hypothalamus, 8–24% exon inclusion) (Supplementary Figure S2). Therefore, this exon must be under strong selection to be skipped most of the time. With this in mind, we examined E24a for any unique sequence features relative to other constitutive and alternative exons of CASK that may drive this skipping. When the splice sites across CASK were computationally scored, we noticed that the 5′ splice site of E24a was not only weaker than those of the constitutive exons (Supplementary Figure S3), but this low score held even when compared to alternative exons E18 and E19 (Figure 3(a)). The E24a 5′ splice site score was 20-fold weaker than that of E18 and 13-fold weaker than that of E19, while the 3′ splice site score was within a 2-fold range of each.

We transplanted the noncanonical 5′ splice site of E24a into a strong constitutive exon, E5 of the mouse APPL2 gene, to test its effect on exon inclusion in a different splicing reporter context (DIP splicing reporter). E5 was chosen as the test exon based on its strong splice sites and similarity in size to CASK E24a. To transplant the 5′ splice site of E24a into this context, two point mutations were made in the 5′ splice site of APPL2 E5 (at positions −2 of the exon and +4 of the intron). These mutations are predicted to significantly weaken base pairing to U1 snRNA (Figure 3(b)). Consistent with this prediction, the mutations produced a dramatic increase in exon skipping (Figure 3(c)). That is, 100% exon inclusion seen with the wild type reporter, DIPwt, was lowered to 35% inclusion with the mutant reporter, DIP5pss. Thus, the E24a 5′ splice site itself confers a strong exon-skipping phenotype in this context.

**Genetic Depletion of SC35 and ASF/SF2 Results in an Increase in E24a Inclusion**

**Figure 4:**
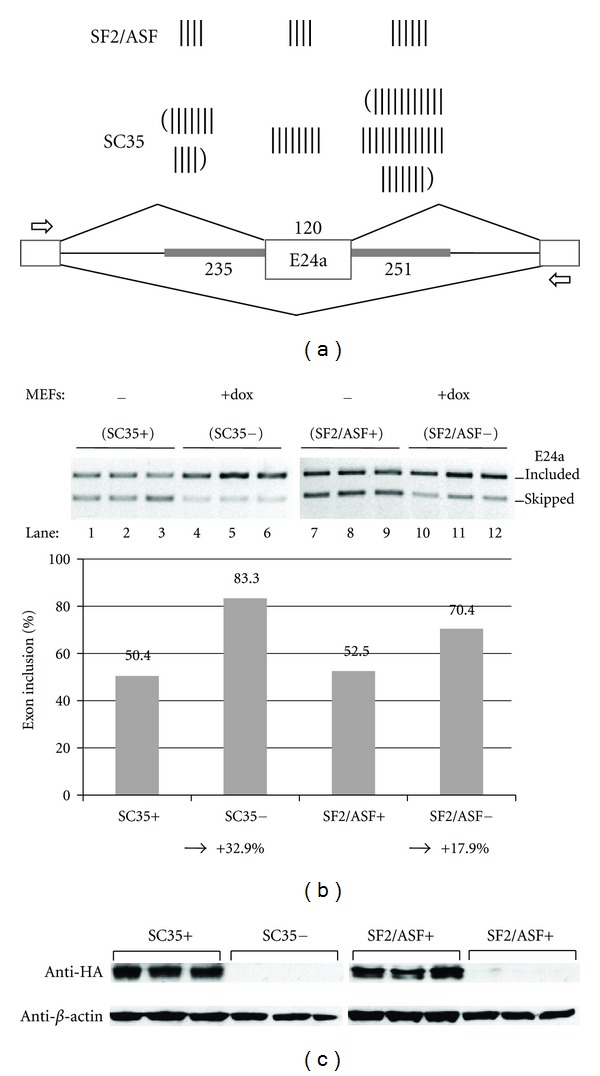
SC35 and ASF/SF2 are negative regulators of CASK E24a. (a) Schematic illustrates the copy number of ASF/SF2 and SC35 motifs in each exon ± ~250 bases of flanking intron. The CASK_E24a splicing reporter is shown below with exons represented as boxes and introns as lines. E24a is inserted as the middle exon with flanking intron sequences (thick grey lines); nucleotide lengths for each segment are indicated. Open arrows indicate primer positions in exons 1 and 3 that were used for the amplification of exon included and skipped products. (b) The CASK_E24a splicing reporter was transfected into MEF cells engineered with an inducible HA-tagged SC35, or ASF/SF2 expression cassette. Gel panels show splicing patterns after cells were cultured in medium containing Dox, which leads to depletion of SC35 (lanes 4–6) or ASF/SF2 (lanes 10–12). Control cells retain SC35 (lanes 1–3) or ASF/SF2 (lanes 7–9) expression when cultured without Dox. Each lane represents an individual transfection experiment. Graph illustrates the average % exon inclusion values for the gel panels shown. Numbers at bottom of graph indicate net change in splicing pattern (c) Depletion of SC35 and ASF/SF2 were verified by Western blotting with an antibody specific for the HA tag. *β*-actin, loading control.

The first hint of an unusual arrangement of SC35 motifs in the neighborhood of E24a (exon ± 250 bases of flanking introns) came from an unbiased analysis of trinucleotide motifs across the entire CASK gene. These results showed enrichment for TGC, CTG, and GCT motifs, which are arranged as tandem repeats in the 250-base pair intron segment just downstream of E24a. These repeats are largely conserved in human and mouse. Upon closer inspection, these sequence repeats were found to coincide with cognate recognition motifs for the splicing factor, SC35 (Supplementary Figure S4). The unusual density of SC35 motifs in the neighborhood of E24a is illustrated graphically in Figure 4(a). Motifs of ASF/SF2, a related member of the SR protein family, are also shown for comparison. Of the 50 SC35 motifs located within E24a and ±250 bases of upstream and downstream flanking introns, 8 motifs are located in the exon itself, 11 in the upstream intron, and 31 with a particularly high density in the downstream intron. There are relatively fewer ASF/SF2 motifs in this region (*n* = 14), and these are more evenly distributed in the exon and flanking intron regions. Far fewer SC35 motifs were found in the neighborhood of E18 (*n* = 21) and E19 (*n* = 11), with similar densities of ASF/SF2 motifs.

To test SC35 as a candidate factor for the regulation of CASK E24a alternative splicing, we took advantage of an inducible SC35 knockout mouse embryo fibroblast (MEF) cell line. In this system, the endogenous SC35 gene has been deleted and the cells complemented with exogenous, HA-tagged SC35 under the control of an inducible Tet-off promoter [33]. Whereas MEF cells grown in the absence of Doxycycline (Dox) express normal levels of HA-tagged SC35, this protein expression is depleted by the addition of Dox. When the CASK_E24a splicing reporter containing mouse E24a middle exon and ~250 bases of adjacent upstream and downstream introns was transfected into the MEF cells in the presence of Dox (SC35−), the inclusion level of E24a was 83%, and in the absence of Dox (SC35+) this level was reduced to 50% (Figure 4(b), lanes 1–6). For comparison, we tested the splicing reporter in a related, ASF/SF2 inducible MEF cell line. These results show 70% exon inclusion in the presence of Dox (ASF/SF2−), and this was reduced to 53% inclusion without Dox (ASF/SF2+) (lanes 7–12). Depletion of SC35 and ASF/SF2 was verified by Western blotting (Figure 4(c)). These results confirm a strong silencing role for SC35 (change of −32.9%) and a relatively weaker silencing role for ASF/SF2 (change of −17.9%), in good agreement with the arrangement of cognate motifs in the neighborhood of E24a and flanking introns.

**E24a is Subject to Negative Regulation by Splicing Silencers Consistent with Local Sequence Architecture**

To further investigate factors that influence E24a skipping, we identified regions of high sequence homology within E24a and flanking introns with the prediction that regulatory motifs would be conserved across mammalian species. The most highly conserved sequence block containing E24a was determined by sequence alignments of homologous introns from mammalian CASK genes (Supplementary Figure S5). This conserved block, which extends ±50 bases into the flanking introns, illustrates the high-density arrangement and close proximity of SC35 motifs to the E24a splice sites. Here, four SC35 motifs encroach upon the noncanonical 5′ splice site from exonic and downstream intronic positions. Moreover, the optimal branch site at position −22 and the secondary branch site at position −33 in the upstream intron each overlap with SC35 motifs.

**Figure 5:**
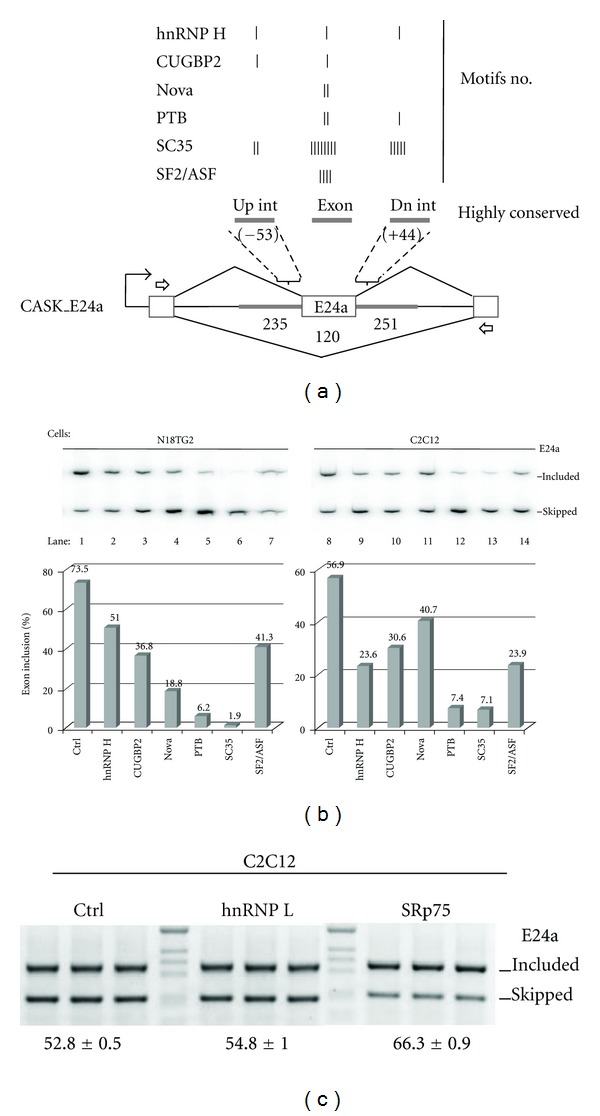
Exon skipping is induced by factors with conserved motifs in close proximity to E24a. (a) The CASK_E24a splicing reporter is shown with the motif copy number for each splicing factor tested in the experiment represented by tick marks in the most highly conserved block corresponding to Supplementary Figure S5: upstream intron (up int), exon, and downstream intron (dn int). (b) Gel panels display the products from splicing reporter assays (E24a included and skipped) in N18TG2 and C2C12 cells. Transfections were performed with protein expression plasmids: hnRNP H (lanes 2, 9), CUGBP2 (lanes 3, 10), Nova (lanes 4, 11), PTB (lanes 5, 12), SC35 (lanes 6, 13), and ASF/SF2 (lanes 7, 14). Control samples (ctrl) were transfected with an equivalent amount of empty vector plasmid (lanes 1, 8). Bar graphs illustrate the % exon inclusion values for the gel panels above. (c) The CASK_E24a splicing reporter was cotransfected into C2C12 cells with an empty vector control (Ctrl), hnRNP L, or SRp75 protein expression vectors. Transfections and RT-PCR amplification from the flanking exons were carried out in triplicate. Percent exon inclusion is indicated below the gel panel, ± standard deviation.

Additional splicing factors have the potential to silence this exon based upon the arrangement of potential regulatory motifs in this highly conserved, uninterrupted sequence block containing E24a. Motifs that have previously been associated with regulation by hnRNP H (AGGG, GGGA), CUGBP2 (GU, UGUGU), Nova (YCAY; Y, pyrimidine), and PTB (UCUU, UCUCU) are located at relative positions that are consistent with silencing roles (Supplementary Figure S5 and Figure 5(a)) [7, 9, 34, 35].

To test these candidate factors for regulation of CASK E24a alternative splicing, the splicing reporter, CASK_E24a, was used in splicing factor coexpression assays in a neuronal (N18TG2) and nonneuronal (C2C12) cell line. In N18TG2 cells, E24a skipping was induced by the coexpression of hnRNP H, CUGBP2, Nova, PTB, SC35, and ASF/SF2 relative to control samples lacking protein expression plasmid (Figure 5(b), lanes 1–7). These factors similarly decreased exon inclusion in C2C12 cells, although the effects were relatively stronger or weaker, indicative of cell-type specific effects (lanes 8–14). For this group of factors, SC35 exerted the most potent silencing effect. These results confirm that a group of splicing silencers promotes E24a skipping, consistent with the local sequence architecture. It is likely that a combination of a weak 5′ splice site, strong silencing by SC35, and combinatorial control by additional splicing silencers results in E24a skipping in most instances.

As additional controls to demonstrate that negative regulation of E24a is specific and not merely a consequence of the weak 5′ splice site, we tested for regulation by hnRNP L, hnRNPA1, and SRp75 in our splicing reporter system. Based upon sequence inspection, we did not expect hnRNP L or hnRNPA1 to regulate this exon. Consistent with this prediction, overexpression of these proteins does not significantly affect the percent exon inclusion of E24a (Figure 5(c) and data not shown). Additionally, we found that SRp75 functions as a weak enhancer of E24a resulting in a 13.5% increase in exon inclusion. The SRp75 binding sites are less well characterized; however it has previously been shown to recognize exonic GA-rich sequence elements for splicing enhancement [36]. Interestingly, a GA-rich cluster is located in the first half of E24a, overlapping potential SC35, ASF/SF2, and hnRNP H silencing motifs. Furthermore, SRp75 is enriched in the central nervous system [37]. Taken together, SRp75 may compete with splicing silencers in a tissue-specific manner to increase the likelihood of E24a inclusion in the brain and spinal cord.

**Mutations in the Downstream Intron Disrupt Splicing Silencing by ASF/SF2 But Not SC35**

To test whether one of the control elements in close proximity to the splice sites might play a role in the silencing effect of SC35, a variety of single mutations were constructed in the CASK_E24a splicing reporter to disrupt SC35 motifs in the branch site and 5′ splice site regions of E24a. None of these single mutations, however, dramatically lessened the silencing effect of SC35 in splicing reporter assays (data not shown). One possible interpretation is that SC35 exerts its silencing effect through multiple control elements acting on an unusually weak 5′ splice site.

**Figure 6:**
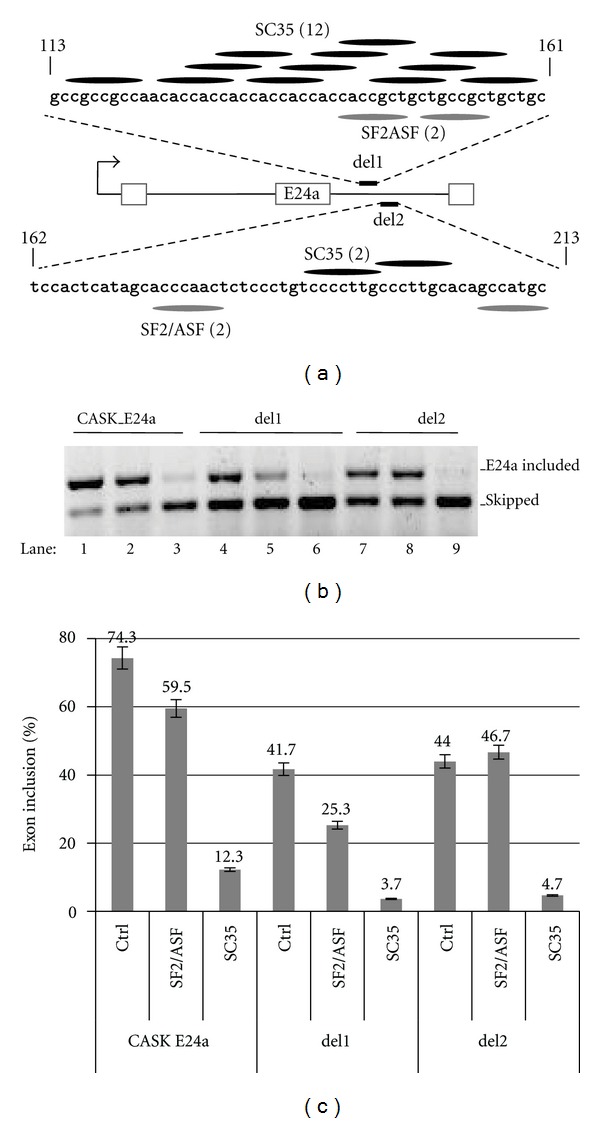
The splicing silencing roles of SC35 and ASF/SF2 are differentially affected by the deletion of cognate motifs in the downstream intron. (a) Schematic indicates structure of parent reporter, CASK_E24a, and derivatives, del1 and del2. The deleted sequences are shown above and below the schematic, where nucleotide positions specify the boundaries of each deletion relative to the beginning of the intron. SC35 motifs, black ovals; ASF/SF2, grey ovals. The copy number of motifs in each deleted region is indicated in parentheses. (b) Gel panel displays exon included and skipped products measured by RT-PCR from N18TG2 transfections. Splicing reporters were cotransfected with ASF/SF2 (lanes 2, 5, 8) or SC35 (lanes 3, 6, 9) expression vector. Control samples (ctrl) received empty vector plasmid (lanes 1, 4, 7). (c) Bar graph illustrates the % exon inclusion values for the gel panel above. Error bars represent standard deviations.

We next explored the effect of deleting the region with the highest density of SC35 motifs in the downstream intron. Deletion 1 (del1) contains 12 SC35 and 2 ASF/SF2 motifs, whereas deletion 2 (del2) contains 2 SC35 and 2 ASF/SF2 motifs (Figure 6(a)). In spite of these differences in the copy number of cognate motifs, SC35 retained an efficient silencing role with the del1 and del2 splicing reporters (Figure 6(b), lanes 3, 6, 9). In contrast, these deletions had differential effects on regulation by ASF/SF2. That is, del1 had little or no effect, and del2 essentially abolished the silencing effect of ASF/SF2 on E24a (lanes 2, 5, 8). In control samples, the effect of each deletion in the absence of SC35 and ASF/SF2 was to decrease the basal level of exon inclusion (from 74% in wild type CASK E24a to 42 and 44% in del1 and del2, resp.) indicating potential roles for these intronic regions in splicing enhancement. Thus, ASF/SF2 likely exerts its silencing role through motifs located in the del2 region of the downstream intron. Unlike ASF/SF2, the exon silencing effects of SC35 cannot be pinpointed to sequences within the del1 or del2 regions of the downstream intron.

## 4. Discussion

We detected a novel exon in the mouse CASK gene based on its unusual exon inclusion behavior in response to neuronal stimulation and NMDA receptor signaling. The exon resides in the most highly conserved sequence block within intron 24 of the Ensembl transcript CASK-001 and is specific to mammals. Hence we designate the name, E24a. While the exon/intron organization of the CASK gene is conserved across vertebrate species, the appearance of E24a during evolution corresponds to *a* > 3,000 base pair jump in intron size from lower vertebrates (lizard, chicken, *Takifugu*, xenopus, zebrafish) to mammals (horse, dolphin, cow, mouse, rat, chimp, human, armadillo).

Under the experimental conditions used here, the induced inclusion of E24a appears to represent a unique splicing response for this exon. By calibrating the KCl-induced splicing pattern change against the natural trend seen during development, we found that stimulation redirected splicing in the direction of, and overshooting, the embryonic pattern. Therefore, it is likely that cell depolarization causes a specific cellular response that results in increased E24a inclusion. In general, there are significant gaps in our understanding of how splicing patterns can be induced to change in response to cellular stress or stimulation [38–41]. Furthermore, little is known about the mechanisms of KCl-induced exon inclusion. Therefore, it would be of interest to dissect the underlying mechanisms of splicing responsiveness of E24a in the future.

Curiously, E24a exhibits composite characteristics of a functional coding exon as well as that of a pseudo-, or false, exon. On the functional side, splicing accuracy and the inclusion of the exon in full-length CASK transcripts were verified. Moreover, the high conservation of the nucleotide sequence and protein coding capacity in mammals indicates the potential biological significance of the exon. On the other side, the low frequency of inclusion of E24a in CASK transcripts, which is generally no higher than 10–15% in the postnatal or embryonic brain, respectively, is a peculiar feature. A related issue is the low functionality of the adjacent 5′ splice site. Our experiments verify the conversion of a constitutive exon to a predominantly skipped exon as a result of transplanting the 5′ splice site of E24a. Previous studies have shown that pseudoexons are associated with a low frequency of exon inclusion, one or more defective splice sites, and the presence of splicing silencer elements [42, 43]. Splicing reporter assays shown here also document splicing silencing of E24a by a variety of tissue-specific and general splicing factors, which indicates that the prevalence of exon skipping may be reinforced by redundant mechanisms. From an evolutionary perspective, it is of interest that new exons emerging from the exonization of intron sequences tend to share these same characteristics [44–46]. Also relevant to our results is the finding from genomewide studies that the birth of new exons in mammals is favored with increasing intron length [47]. This also fits with the substantial jump in intron length for mammalian introns where E24a sequences are found.

We identify a high density of SC35 motifs as an unusual feature of the sequence architecture in the neighborhood of the exon. Consistent with this observation, we show that SC35 induces strong exon skipping of E24a when overexpressed, which is reversed when SC35 is depleted. SC35 is a well-studied splicing factor of the SR protein family with roles in the kinetic commitment of a pre-mRNA to the splicing pathway and in the regulation of splice site selection [48, 49]. Aside from their enhancing roles during early spliceosome assembly, SR proteins can act as splicing silencers when their cognate motifs are located in introns [50]. A well-documented example of a role for SC35 in 5′ splice site silencing is found in the E1 alpha subunit of the pyruvate dehydrogenase gene. In this case, an intronic point mutation generates a de novo SC35 motif just downstream of the natural 5′ splice site, inhibiting the use of the splice site and leading to a protein defect associated with mental retardation [51, 52]. Furthermore, a mutation that generates a new exonic SC35 motif has been linked to pathological E3 skipping in the human growth hormone gene [53]. In this study, we were unable to pinpoint the exact motifs for SC35 regulation of CASK E24a, which likely reflects the use of redundant sequence elements for exon skipping.

In addition to the silencing effect mediated by SC35, we verify that ASF/SF2 induces exon skipping of E24a through a cluster of downstream intronic motifs and demonstrate that hnRNP H, CUGBP2, Nova, and PTB exert silencing effects consistent with the arrangement of highly conserved motifs in this neighborhood. Furthermore, we show that the brain-enriched SR protein, SRp75, induces exon inclusion, suggesting a tissue-specific role for this factor. SR proteins are subject to dynamic regulation by phosphorylation events, which we speculate may be key to the modification of their activities by signaling pathways [54]. In this case, individual or combined modifications of SRp75, SC35, and/or ASF/SF2 may contribute to the splicing change observed under depolarizing conditions. Consistent with this hypothesis, altered levels of SC35 expression have been implicated in the stress-induced alternative splicing of acetylcholinesterase transcripts [55]. Furthermore, the splicing silencing function of ASF/SF2 bound to an intronic element has been shown to be sensitive to dephosphorylation by protein phosphatase 2A [56].

**Figure 7:**
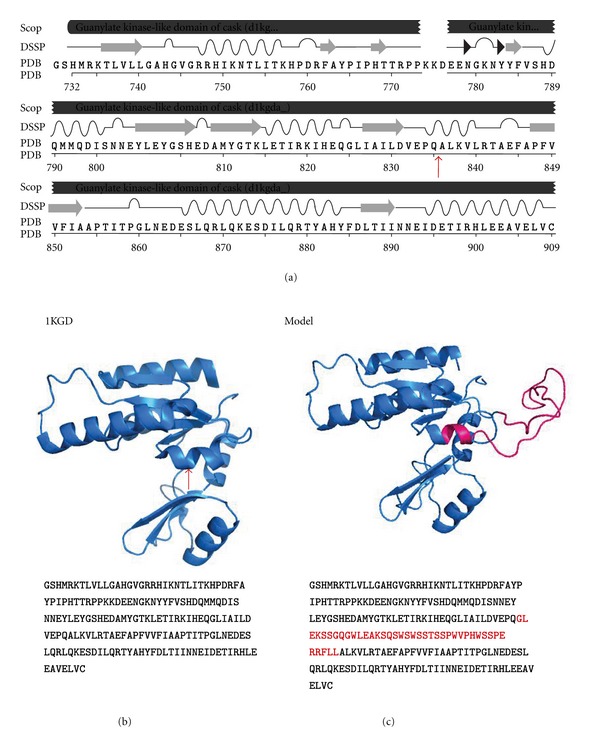
E24a codes for a peptide insertion in the guanylate kinase domain of the CASK polypeptide. (a) Sequence of human guanylate kinase domain according to pdb structure, 1KGD, with secondary structure elements indicated as follows: arrows, beta strands; coils, alpha helices. Position of E24a-encoded peptide insertion is between residues Q835 and A836 (vertical arrow). (b) PyMOL representation of guanylate kinase domain from pdb structure, 1KGD [31]. Secondary structure elements are shown as in (a). The position of the E24a peptide insertion is indicated (vertical arrow). (c) The E24a peptide insertion was threaded onto the CASK guanylate kinase-like domain (1KGD) using MUSTER [32]. The E24a insertion is shown in pink.

The exact role of E24a is not understood. The roles of the CASK polypeptide at the developing synapse and in the regulation of neuronal gene expression are mediated through a large variety of protein-protein interactions. Our results predict that the inclusion of E24a should lead to the insertion of a 40 amino acid peptide module in the middle of the guanylate kinase domain (Figure 7(a), red arrow). The guanylate kinase domain is a pseudokinase involved in interactions with the transcription factor, TBR-1, and the nucleosome assembly factor CASK-interaction nucleosome assembly protein (CINAP). A protein complex including CASK, TBR-1, and CINAP accumulates in the nucleus and regulates the transcription of genes important for neuronal function and development, such as RELN and the NMDA receptor subunit 2b [22, 57, 58]. The crystal structure of the guanylate kinase domain of human CASK reveals a globular tertiary fold with GMP-binding, lid, and core regions (Figure 7(b), PDB file; 1KGD) [31]. Modeling of the human guanylate kinase domain containing the E24a peptide insertion using 1KGD as the template demonstrates that this insert has the potential to maintain domain structure with the addition of an extended loop that may alter the protein interaction properties of this domain (Figure 7(c)). This loop is quite hydrophobic and contains low complexity sequence and is therefore likely flexible and unstructured. Furthermore, several serine residues provide the potential for posttranslational modification of this loop, which may alter the localization or activity of the protein. Future studies will be needed to address the question of whether the peptide insertion diversifies the function of the guanylate kinase domain, alters protein-protein interaction properties or cellular localization of the protein, or serves as a neutral appendage. CASK is a candidate gene for X-linked mental retardation and eye movement disorders. Our results suggest that an understanding of its inducible splicing regulation may provide new leads for the identification of potential modifiers of its polypeptide activities.

## Supplementary Material

Supplementary Figure S1. Analysis of splicing patterns in mouse cerebral cortex for CASK E18, E19, and E24a at distinct developmental stages.
Supplementary Figure S2. Tissue-specific expression of CASK E24a.
Supplementary Figure S3. Comparison of relative MAXENT splice site z-scores across the CASK gene.
Supplementary Figure S4. Relative SC35 motif copy number in intron segments of CASK.
Supplementary Figure S5. The non-canonical 5*´* splice site is situated in a high-density environment of splicing silencing motifs within the most highly conserved sequence block containing E24a.

## Figures and Tables

**Table 1:** CASK E24a is associated with peptide coding capacity in mammals.

Species	E24 (bp)	Intron 24 (bp)	E25 (bp)	E24a^‡^ identity	E24a encoded peptide
Horse	203	10159	84	100%	GLEKSSGQGWLEAKSQSWSWSSTSSPWVPHWSSPERRFLL
Dolphin	203	10066	84	98.3%	GLEKSSGQGWLEEKSQPWSWSSTSSPWVPHWSSPERRFLL
Cow	203	8084	84	98.3%	GLEKSSGQGWLEAKSQSWSWSSTPCPWVPHWSSPERRFLL
Mouse	203	7258	84	self	GLEKSSGQGWLEAKSQSWSWSSTSSPWVPHWSSPERRFLL
Rat	203	7239	84	100%	GLEKSSGQGWLEAKSQSWSWSSTSSPWVPHWSSPERRFLL
Chimp	203	7143	84	100%	GLEKSSGQGWLEAKSQSWSWSSTSSPWVPHWSSPERRFLL
Human	203	6972	84	100%	GLEKSSGQGWLEAKSQSWSWSSTSSPWVPHWSSPERRFLL
Armadillo	203	6207	84	100%	GLEKSSGQGWLEAKSQSWSWSSTSSPWVPHWSSPERRFLL
Lizard	203	2878	84	—	—
Chicken	203	2809	84	—	—
Takifugu	203	1180	84	—	—
Xenopus	203	641	84	—	—
Zebrafish	203	252	84	—	—

E24, intron 24, and E25 lengths (base pairs, bp) for orthologous CASK genes using the exon numbers from mouse Ensembl transcript CASK-001. ^‡^Nucleotide sequence identities to mouse E24a as determined by ClustalX alignments of orthologous intron regions. Peptide sequences encoded by E24a (differences from mouse are underscored) are shown in far right column. Dashes indicate the absence of E24a or its encoded peptide in non-mammalian species.
