# Supplementary material for: Alternative Splicing of a Novel Inducible Exon Diversifies the CASK Guanylate Kinase Domain
Source: J Nucleic Acids. 2012 Sep 12;2012:816237. doi: 10.1155/2012/816237 (PMC3447378; doi:10.1155/2012/816237)
Supplement: Supplementary file 1 — Supplementary Figure S1. Analysis of splicing patterns in mouse cerebral cortex for CASK E18, E19, and E24a at distinct developmental stages. Supplementary Figure S2. Tissue-specific expression of CASK E24a. Supplementary Figure S3. Comparison of relative MAXENT splice site z-scores across the CASK gene. Supplementary Figure S4. Relative SC35 motif copy number in intron segments of CASK. Supplementary Figure S5. The non-canonical 5´ splice site is situated in a high-density environment of splicing silencing motifs within the most highly conserved sequence block containing E24a. [file 816237.f1.pdf]

Supplementary Figure S1

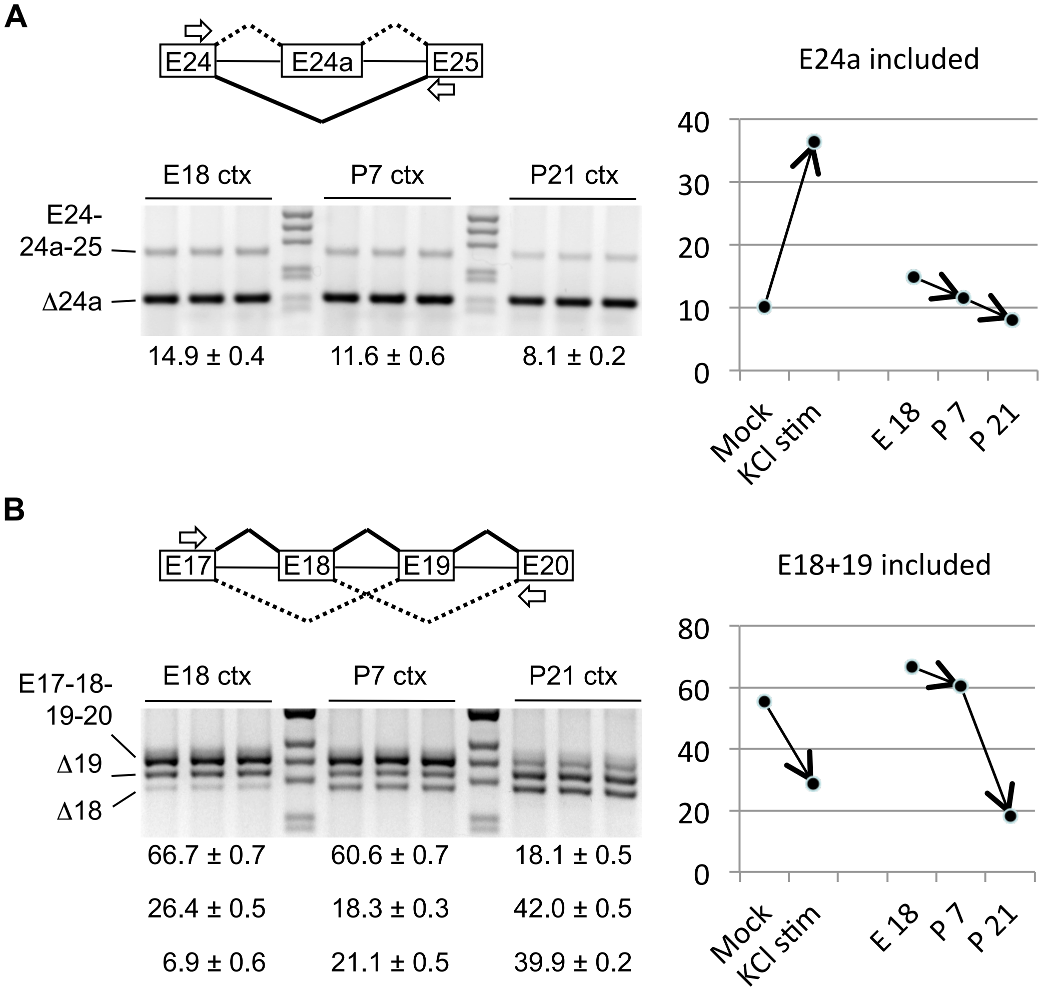

Analysis of splicing patterns at distinct developmental stages reveals a decrease in E18, E19 and E24a inclusion with mouse development. (A) Gel panel shows triplicate measurements for E24a included (E24-24a-25) and skipped (Δ24a) products for embryonic day 18 (E18 ctx), postnatal day 7 (P7 ctx) and 21 (P21 ctx) cerebral cortex using PCR primers as indicated in schematic. Lanes 4 and 8, 1 kb DNA ladder. Numbers below panel represent average % exon inclusion ± standard deviation. Graph displays % exon inclusion values (y-axis) for KCl stimulation and developmental series with arrows representing the overall effect on exon inclusion. (B) Same as (A), but for exons E18 and E19.
