# Supplementary material for: Alternative Splicing of a Novel Inducible Exon Diversifies the CASK Guanylate Kinase Domain
Source: J Nucleic Acids. 2012 Sep 12;2012:816237. doi: 10.1155/2012/816237 (PMC3447378; doi:10.1155/2012/816237)
Supplement: Supplementary file 2 [file 816237.f2.pdf]

Supplementary Figure S2

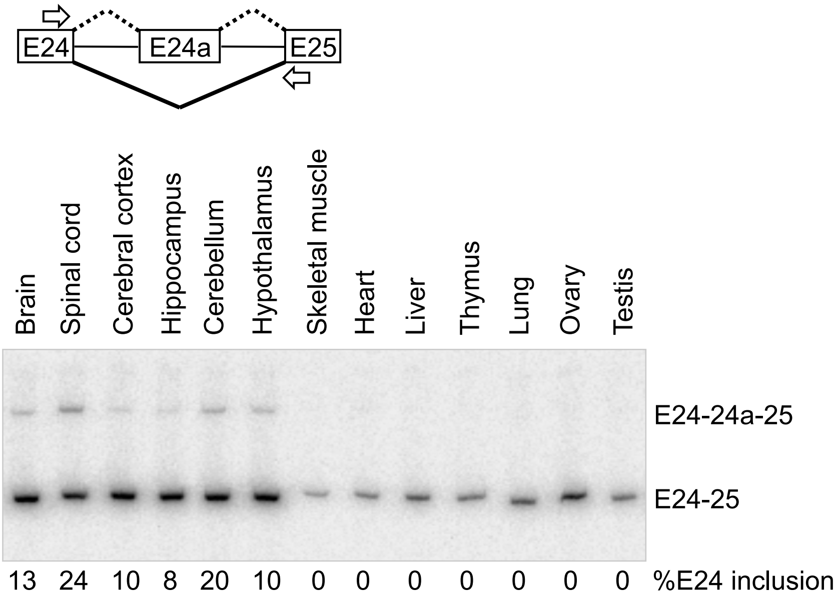


**Tissue-specific expression of CASK E24a.** RNA was isolated from adult mouse tissue as indicated and subjected to RT-PCR amplification using primers specific for E24 and E25 as shown in the schematic above. Percent E24a exon inclusion for individual cell types is indicated below the gel.
