# Supplementary material for: Alternative Splicing of a Novel Inducible Exon Diversifies the CASK Guanylate Kinase Domain
Source: J Nucleic Acids. 2012 Sep 12;2012:816237. doi: 10.1155/2012/816237 (PMC3447378; doi:10.1155/2012/816237)
Supplement: Supplementary file 3 [file 816237.f3.pdf]

Supplementary Figure S3

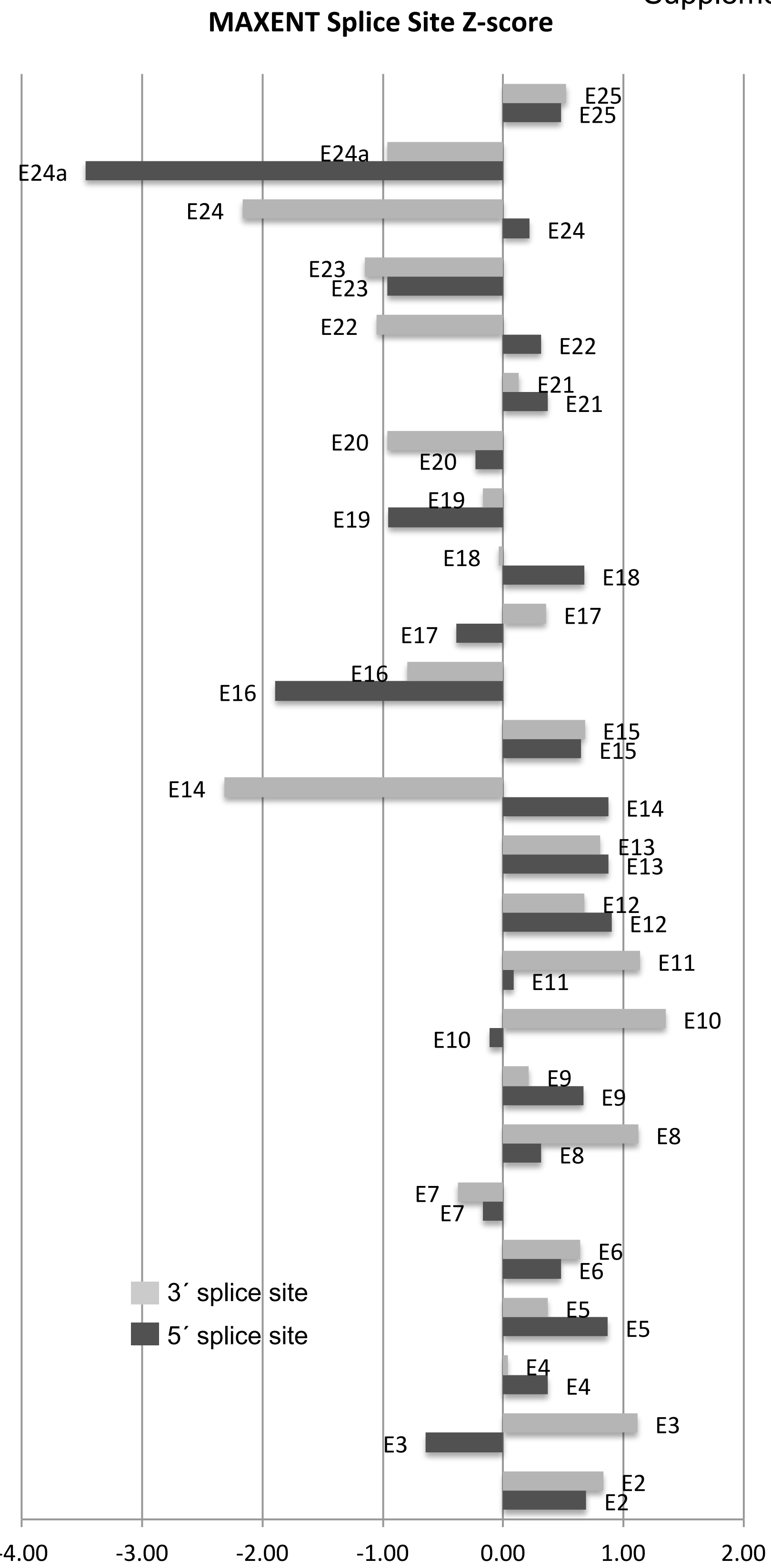

Comparison of relative MAXENT splice site z-scores across the CASK gene. Relative z-scores were calculated for the 3´ (light grey) and 5´ (dark grey) MAXENT splice site values of all annotated exons of the CASK gene in addition to the newly identified E24a. Negative z-scores indicate splice sites with values less than the mean and positve z-scores indicate splice sites with values greater than the mean.
