# Supplementary figures and images for: Alternative Splicing of a Novel Inducible Exon Diversifies the CASK Guanylate Kinase Domain
Source: J Nucleic Acids. 2012 Sep 12;2012:816237. doi: 10.1155/2012/816237 (PMC3447378; doi:10.1155/2012/816237)

Supplementary Figure S4

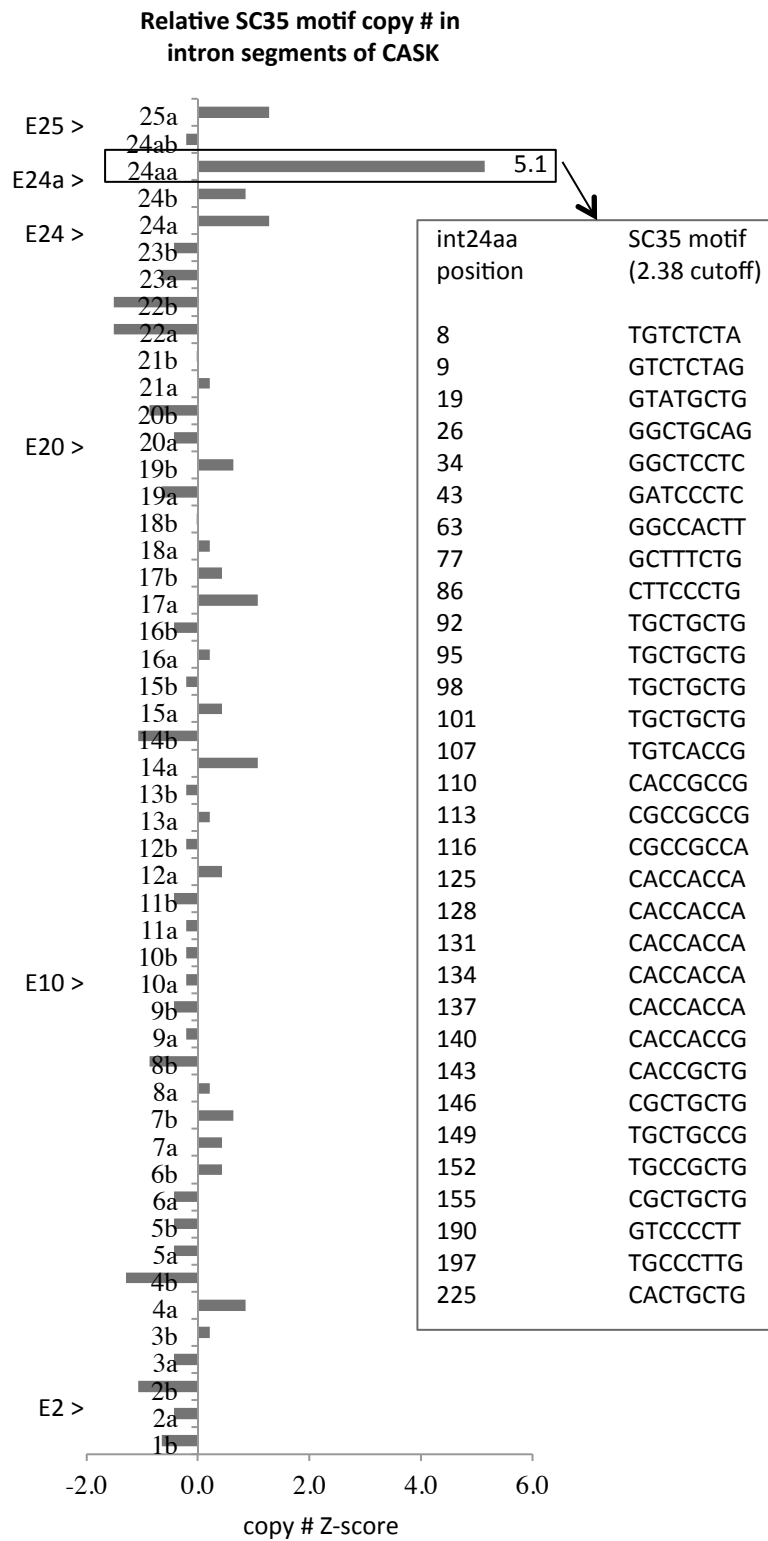

Supplement: Supplementary file 4 [file 816237.f4.pdf]
