# Supplementary material for: Alternative Splicing of a Novel Inducible Exon Diversifies the CASK Guanylate Kinase Domain
Source: J Nucleic Acids. 2012 Sep 12;2012:816237. doi: 10.1155/2012/816237 (PMC3447378; doi:10.1155/2012/816237)
Supplement: Supplementary file 5 [file 816237.f5.pdf]

Supplementary Figure S5

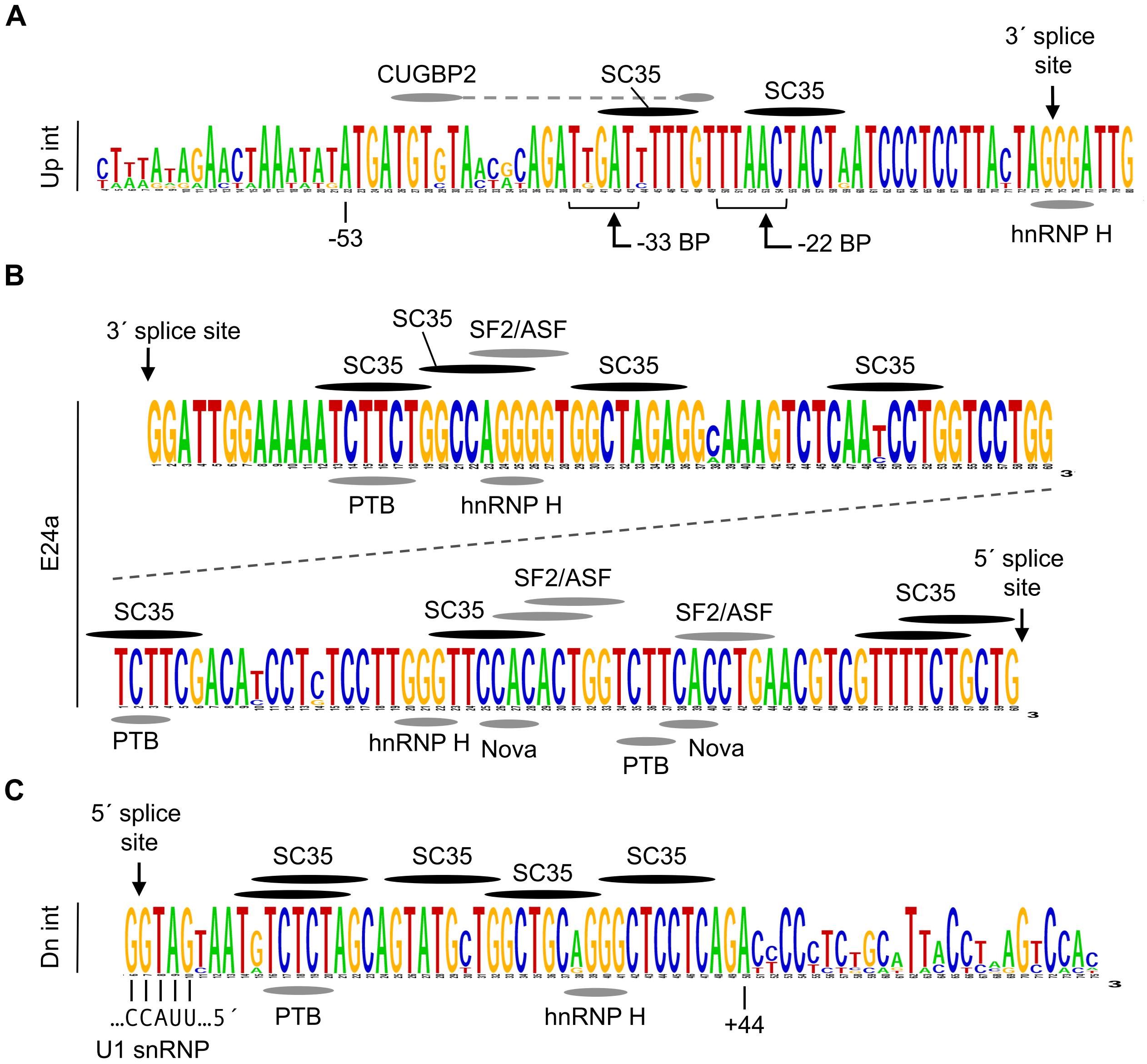

The non-canonical 5´-splice site is situated in a high-density environment of splicing silencing motifs within the most highly conserved sequence block containing E24a. A multiple sequence alignment for E24a and flanking introns is displayed in a Weblogo format (weblogo.berkeley.edu). Homologous intron 24 sequences were taken from horse, dolphin, cow, mouse, chimp, human and armadillo CASK genes using the Ensembl database. The most highly conserved block of sequence extending from position -53 in the upstream intron to +44 of the downstream intron: (A) upstream intron, (B) exon, (C) downstream intron. Arrows indicate 3´ and 5´ splice sites. Vertical lines indicate base pairing of the 5´ splice site to U1 snRNA. Candidate branchpoints (BP) at positions -33 and -22 are the only sequences matching the consensus, YUNAY (brackets; Y, pyrimidine; N, any nucleotide) in the region shown. Black ovals represent positions of SC35 motifs; grey ovals signify CUGBP2 (TGTGT, TGT), hnRNP H (AGGG), PTB (TCTT, TCTCT), SF2/ASF, and Nova (YCAY; Y, pyrimidine). SC35 and SF2/ASF motifs were scored with ESEFinder 3.0.
